# Prevalence and risk factors associated with headache amongst medical staff in South China

**DOI:** 10.1186/s10194-020-1075-z

**Published:** 2020-01-14

**Authors:** Wei Xie, Ruibing Li, Mianwang He, Fang Cui, Tingting Sun, Jianmei Xiong, Dengfa Zhao, Weinan Na, Ruozhuo Liu, Shengyuan Yu

**Affiliations:** 1 International Headache Center, Department of Neurology, the First Medical Center, Chinese PLA General Hospital, Fuxing Road 28, Haidian District, Beijing, 100853 China; 2 Department of Laboratory Medicine, the First Medical Center, Chinese PLA General Hospital, Fuxing Road 28, Haidian District, Beijing, 100853 China; 3 Department of Neurology, Hainan Hospital of Chinese PLA General Hospital, Jianglin Road 28, Sanya, 572013 China

**Keywords:** Prevalence, Headache, Migraine, Tension-type headache, Medical staff

## Abstract

**Background:**

A previous study by our team reported the prevalence of primary headache disorders and factors associated with headache among nurses in three hospitals in North China. The aim of this cross-sectional survey was to learn more about how medical nurses in South China were affected by headache. Additionally, we determined the prevalence of headache and measured the impact of headache among doctors in mainland China for the first time.

**Methods:**

Stratified random cluster sampling was used to select 280 physicians and 365 nurses from various departments in four hospitals in Sanya, which is one of southernmost cities in China. Information was collected on demographic data, occupational factors and headache characteristics by using a structured questionnaire.

**Results:**

Among 645 medical staff, 548 (85%) responded (doctors = 240, nurses = 308). Among the medical staff, the 1-year prevalence of primary headache disorders was 50%, with 25.9% experiencing migraine and 24.1% experiencing tension-type headache (TTH). The prevalence of migraine in female doctors was higher than that in female nurses, although this difference was not significant (32.4% vs. 29.8%, *P* = 0.628). Multivariate analysis showed that being female and working in other specialties (Emergency Department & Radiology Department) remained independent risk factors for migraine in doctors (OR 2.314 and 3.223). In nurses, being married was a risk factor for migraine (OR 3.728), and job titles remained an independent risk factor for migraine and TTH (OR 2.294 and 4.695). Working more than 6 night-shifts per month was associated with an increased prevalence of migraine and TTH in doctors; the same was true in nurses for migraine, but not for TTH.

**Conclusion:**

The prevalence of primary headache disorders in both nurses and doctors is higher than that in the general population in South China. Our study shows that occupation, geography and sex may play an important role. Further, female doctors are more susceptible than female nurses to migraine. The risk factors relevant to headache that were found in this study should provide an important reference for promoting occupational health in medical staff, especially female doctors in China.

## Background

Few people never experience headache in their lifetime due to the high prevalence of this disorder in the general population throughout the world. The most prevalent headache disorders are migraine and tension-type headache (TTH) [1]. The 2016 Global Burden of Disease (GBD) study established TTH as the third most prevalent condition in the world and migraine as the sixth [2]. A population-based study in China showed that the 1-year prevalence of primary headache disorders was 23.8% [3], which appears to be much lower than that in other countries and regions. In other Asian countries, the 1-year prevalence of headache disorders ranged from 64%(India) to 85%(Nepal) [4]. European regional studies showed that 53%–75%of people experienced headache disorders, while the 1-year headache prevalence was 45% in Ethiopia and 62% in Zambia [4]. Despite the differences in prevalence in different regions and countries, the socioeconomic impact of headache disorders was similar: The overall lost gross domestic product (GDP) was approximately 1.7% [4]. Headache can substantially impair quality of life and is ranked sixth among the leading causes of years lived with disability (YLDs) worldwide [5, 6].

Some epidemiological studies have shown that several risk factors, including sex [7], body mass index (BMI) [8], smoking [9], family history [10], and climate [11] might be associated with primary headache disorders. Despite the lower prevalence in China, the risk factors are similar to those reported for other countries [12–14]. In a previous study in North China, we found that the prevalence of primary headache disorders in nurses was significantly higher than that in the general population (45.3% vs. 23.8%) [15]. Occupational factors may play a vital role in the pathogenesis of headache. Medical staff, including nurses, confront heavy workloads and experience states of high tension and high intensity for long periods [15]. Therefore, more attention should be paid to the health status of medical staff. In addition to occupation, climate affects the development of headache to varying degrees [16, 17]. China is a vast country spanning the temperate zone, tropics and subtropics. Hainan is the only province completely within the tropical region. It is located in the southernmost part of China, and Sanya city is located at the southern tip of Hainan. The headache rate of medical staff in this area is not yet available.

In this paper, we sought to provide recent statistics on the prevalence of primary headache disorders in medical staff in South China. Our aims were to update prior prevalence estimates and to identify other factors relevant to headache disorders among medical staff in China, which should be a supplementary extension of “*Lifting The Burden*” in China.

## Methods

### Ethics

The study protocol was approved by the Ethics Committee of the Chinese PLA General Hospital, Beijing. All patients were informed of the purpose of the study and provided informed consent prior to participating.

### Sample and survey

This is a cross-sectional survey conducted in three tertiary A hospitals (Hainan Hospital of Chinese PLA General Hospital, the Third People’s Hospital of Hainan Province and the People’s Hospital of Sanya) and one secondary A hospital (the Chinese PLA No. 425 Hospital) in Sanya, South China, from May 2018 to October 2018. We conducted a preliminary survey of one department in each hospital to test epidemiological methods and then adopted a stratified random cluster sampling method. In each hospital, we randomly selected eight clinical departments, from which all doctors and nurses were invited to participate the headache survey. The survey was divided into 2 parts: questionnaires and interviews (face-to-face interviews & telephone interviews). All those reporting headache were interviewed after their questionnaires were reviewed by neurologists.

### Procedure

#### Questionnaires

Neurologists, who had been systematically trained with The International Classification of Headache Disorders, 3rd edition (ICHD-3) tool and the survey, explained the notes for the questionnaire and answered the participant’s questions. Then each participant completed the structured questionnaire to gather demographic data, occupation-related factors and headache characteristics over the previous year. The demographic and headache profile sections of the questionnaire were the same items as used in a Chinese national epidemiology study, and were validated for headache assessment and diagnosis in the general population [3, 15, 18].

A.The demographic characteristics section: Sex, Age, Ethnicity (Han v.s. Non-Han), Marital Status: Unmarried (including single and divorced people) v.s. Married, Education (College or lower, Bachelor’s degree, Master’s degree or above), and body mass index (BMI, graded as Underweight, Normal Weight, Overweight, Obese).

B.The occupational characteristics section: Specialty (Internal Medicine, Surgical Department, Others: Emergency Department and Radiology Department), Work Seniority (Doctors: < 7 years v.s. ≥7 years; Nurses: < 5 years v.s. ≥5 years; 5 and 7 were the median), Title (Junior, Senior and Advanced. Doctors: Resident, Attending Physician and Deputy Chief Physician or above; Nurses: Nurse, Nurse Practitioner and Nurse-in-charge or above), Work Arrangements (rotational shifts) and Number of Night Shifts (for those performing shift work).

C.The headache profile section (25 questions): Headache Duration, Frequency, Location, Quality, Intensity, Aura, Characteristics of Accompanying Symptoms (Nausea, Vomiting, Photophobia and Phonophobia) and The Impact of Physical Activity on Headache, etc. Diagnostic questions began with a headache screening question “Have you had a headache in the last year not related to flu, hangover, cold or head injury?”. Those who answered “no” to this question were classified as headache-free. All those reporting headache were invited to interviews after their questionnaires were reviewed. In this questionnaire, trigeminal autonomic cephalalgias (TACs), other primary headache disorders and secondary headaches were not included.

#### Interviews

In face-to-face interviews, trained neurologists would confirm the headache diagnosis based on the ICHD-3. Any participant who might have more than one type of headache was instructed to focus on the subjectively most bothersome type (meaning that only one headache type was diagnosed) for purposes of description, diagnosis and prevalence counting. Participants reporting headache occurs 15 or more days/month for more than 3 months were diagnosed with chronic daily headache (CDH) [1, 19, 20] and questioned if medication-overuse headache (MOH). Neurologists would consider differential diagnoses, and remain alert to other possible diagnosis that raise a need for further examinations: the Red flag mostly to signal cases that might be secondary headache. If necessary, they would perform general and neurological examination.

All the participants were interviewed through telephone, especially for originally headache-free participants and headache participants with ambiguous memories to lower the recall bias.

### Statistics

The statistical analyses were conducted with IBM SPSS (version 23.0). Continuous variables that did not comply with the normal distribution were summarised as medians, and categorical variables were summarised as numbers and percentages. We used Chi-squared to compare distributions of categorical variables between groups, and used Bonferroni correction to adjust the statistical results for multiple comparisons (when the product of the Least Significant Difference *P*-value and the number of comparisons exceeds 1, the Bonferroni-adjusted *P*-value would be set 1.000). Multivariate logistic regression was applied to identify odds ratios (ORs) with 95% confidence intervals (CIs) for different types of headache according to demographic and occupational characteristics. Statistical significance was set at *P* < 0.05.

## Results

Among the 645 medical staff invited to participate, 22 declined to complete the survey, and 75 submitted questionnaires with incomplete or perfunctory responses. The response rate was 85%. A total of 548 respondents completed the survey (doctors = 240, nurses = 308). The age ranged from 20 to 60 years, with a median of 28 years (doctors = 31 years, nurses = 27 years).

Only 3 respondents were diagnosed with two types of primary headache, and 18 had other headaches, including neuralgia, CDH, and unclassifiable headache. Only 1 nurse was diagnosed with MOH. As the number of people suffering from CDH and MOH was low, we did not take these disorders into account.

**Fig. 1:**
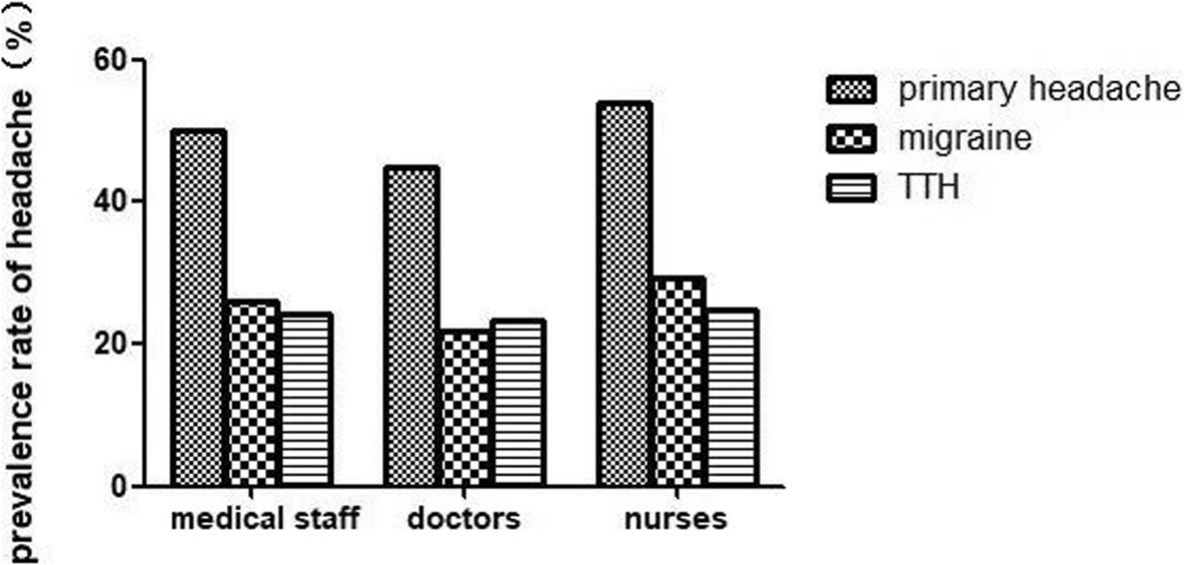
Comparison of the prevalence of different types of headaches among medical staff, doctors and nurses

**Fig. 2:**
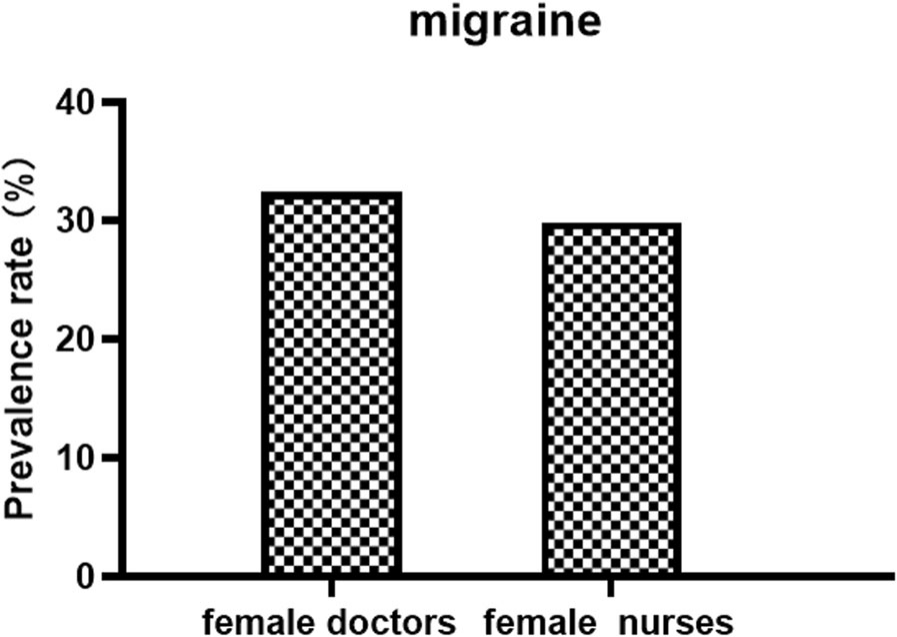
Comparison of the prevalence of migraine among female doctors and female nurses

**Fig. 3:**
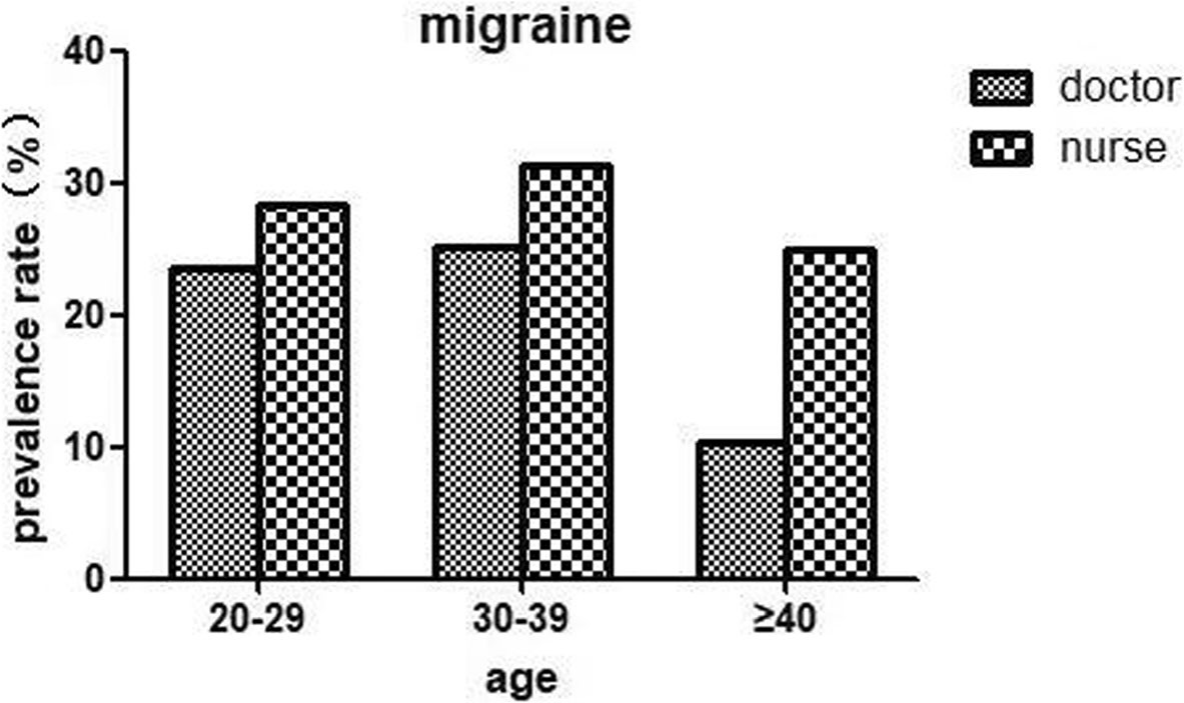
Prevalence of migraine among doctors and nurses by age

**Fig. 4:**
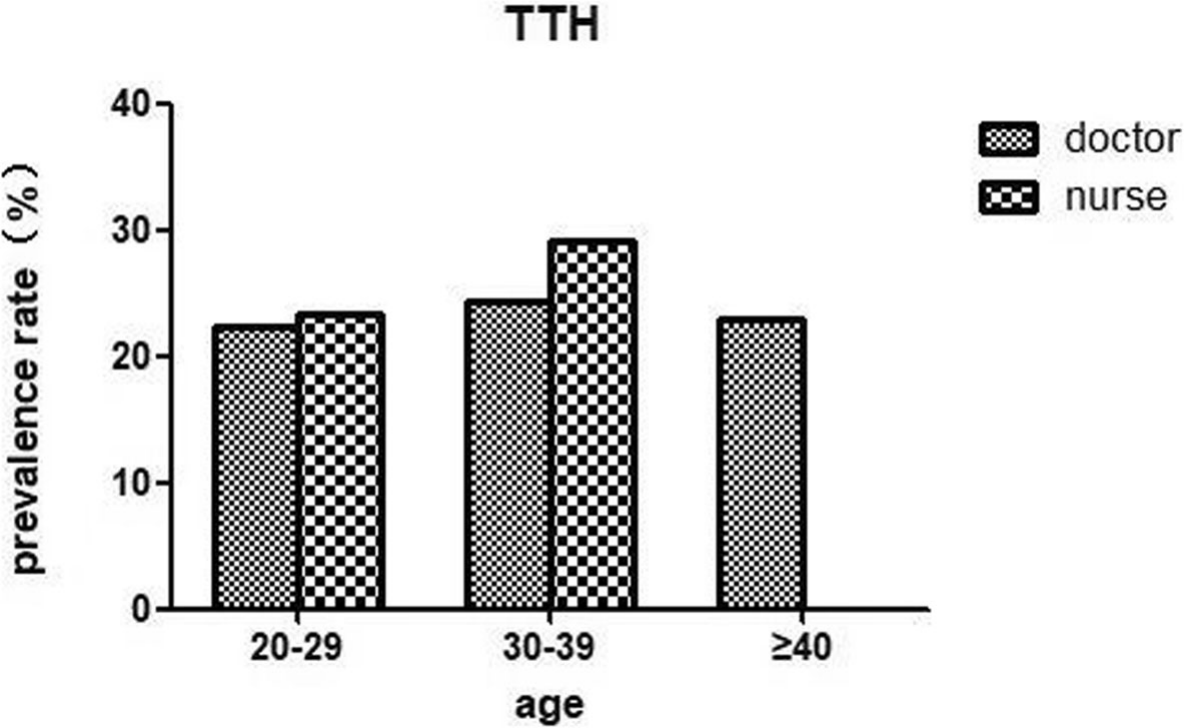
Prevalence of TTH among doctors and nurses by age

Table 1 and Fig. 1 showed that among the medical staff, the 1-year prevalence of primary headache disorders was 50% (95% CI 45.8–54.2%), with 25.9% (95% CI 22.2–29.6%) experiencing migraine and 24.1% (95% CI 20.5–27.7%) experiencing TTH. Both types of primary headache were more prevalent in nurses than in doctors (migraine: 29.2% vs. 21.7%, *P* = 0.045; TTH: 24.7% vs. 23.3%, *P* = 0.715) (showed in Table 2, Fig. 1). However, by further dividing doctors and nurses into two groups by sex, we found that the group of female doctors had the higher prevalence of migraine than female nurses, although this difference was not significant (32.4% v.s. 29.8%, *P* = 0.628) (see Table 2 and Fig. 2). The prevalence of both migraine and TTH peaked during middle age (30–39 years) in the nurse and doctor groups but were higher in the former group than in the latter (migraine: 31.4% vs. 25.2%, *P* = 0.168 < 0.05; TTH: 29.1% vs. 24.3%, *P* = 0.213 < 0.05). Because there were very few nurses over the age of 40 and none of them had TTH, we did not count them (see Figs. 3 and 4).

**Table 1:** The demographic characteristics comparisons between different types of headaches and non-headache among medical staff

Variable	Non-headache	Total headache	Migraine	TTH
	N	N(%) P	N(%) Bonferroni *P*	N(%) Bonferroni *P*
Total	256	292(53.3)	142(25.9)	132(24.1)
Age		0.067	0.072	0.969
20–29	140	163(53.8)	82(27.1)	70(23.1)
30–39	84	109(56.5)	54(28.0)	51(26.4)
≥40	32	20(38.5)	6(11.5)	11(21.2)
Ethnicity		0.421	1.000	0.675
Han	238	266(52.8)	131(26.0)	118(23.4)
Non-Han	18	26(59.1)	11(25.0)	14(31.8)
Marital status		0.051	0.063	1.000
Unmarried (Single&Divorced)	130	124(48.8)	55(21.7)	61(24.0)
Married	126	168(57.1)	87(29.6)	71(24.1)
Education		0.672	0.303	1.000
College or lower	76	97(56.1)	57(32.9)	35(20.2)
Bachelor	151	163(51.9)	70(22.3)	81(25.8)
Master or above	29	32(52.5)	15(24.6)	16(26.2)
BMI		0.982	1.000	1.000
Lower than normal (< 18.5)	44	49(52.7)	22(23.7)	23(24.7)
Normal (18.5- < 23)	128	145(53.1)	80(29.3)	59(21.6)
Overweight (23- < 25)	38	47(55.3)	22(25.9)	20(23.5)
Obese(≥25)	46	51(52.6)	18(18.6)	30(30.9)

**Table 2:** Prevalence of migraine and Tension-type headache among doctors and nurses of different genders

Variable	Doctors				Nurses			
	Non-headache N	Total headahe	Migraine	TTH	Non-headache N	Total headache	Migraine	TTH
		N(%) *P*	N(%) *P*	N(%) *P*		N(%) *P*	N(%) *P*	N(%) *P*
Total	127	113(47.1)	52(21.7)	56(23.3)	129	179(58.1)	90(29.2)	76(24.7)
Sex		0.193	0.002	0.712		0.409	0.241	1.000
male	78	60(43.5)	19(13.8)	36(26.1)	4	2(33.3)	0(0)	2(33.3)
female	49	53(52.0)	33(32.4)	20(19.6)	125	177(58.6)	90(29.8)	74(24.5)

Comparisons of the demographic data for medical staff with different types of headaches and non-headache are shown in Table 1. Univariate analysis suggested that age, marital status, ethnicity, education, and BMI were not linked to either of two types of primary headaches.

Comparisons of the occupation-related data between different types of headaches and non-headaches for doctors and nurses are shown in Tables 3 and 4. Specialty did not correlate with any type of headaches in doctors (migraine: internal medicine: 20.3%, surgical department: 19.2%, other specialties: 34.5%, *P* = 0.786 > 0.05; TTH: internal medicine: 22.5%, surgical department:27.4%, other specialties: 17.2%, *P* = 1.000 > 0.05) or with TTH in nurses (internal medicine: 26.2%, surgical department: 22.8%, other specialties: 26.1%, *P* = 1.000 > 0.05). However, internal medicine nurses were less likely to experience migraine than nurses in the surgical department (internal medicine: 21.5%, surgical department: 36.0%, *P* = 0.033 < 0.05). The prevalence of TTH was significantly higher for some nursing roles (nurse: 15.6%, nurse practitioner: 34.0%, *P* < 0.001), but this was not observed for doctors (resident: 22.5%, attending physician: 26.6%, deputy chief physician or above: 21.3%, *P* = 1.000 > 0.05). The prevalence of migraine and total headache did not significantly differ with title for either doctors or nurses. Seniority and work arrangement had no effect on the prevalence of headache, including migraine and TTH, in doctors or nurses.

**Table 3:** The occupational characteristics comparisons between different types of headache and non-headache among doctors

Variable	Non-headache N	Total headache	Migraine	TTH
		N(%) *P*	N(%) Bonferroni *P*	N(%) Bonferroni *P*
Total	127	113(47.1)	52(21.7)	56(23.3)
Specialty		0.505	0.786	1.000
Internal Medicine	77	61(44.2)	28(20.3)	31(22.5)
Surgical Department	37	36(49.3)	14(19.2)	20(27.4)
Others	13	16(55.2)	10(34.5)	5(17.2)
Seniority (year)		0.945	1.000	1.000
< 5 or 7	59	53(47.3)	27(24.1)	24(21.4)
≥5 or 7	68	60(46.9)	25(19.5)	32(25.0)
Title		0.136	0.222	1.000
Junior	64	65(50.4)	33(25.6)	29(22.5)
Senior	32	32(50.0)	14(21.9)	17(26.6)
Advanced	31	16(34.0)	5(10.6)	10(21.3)
Work arrangement		0.419	0.249	1.000
Day-shift	35	26(42.6)	8(13.1)	16(26.2)
Rotating-shift	92	87(48.6)	44(24.6)	40(22.3)

**Table 4:** The occupational characteristics comparisons between different types of headache and non-headache among nurses

Variable	Non-headache N	Total headache	Migraine	TTH
		N(%) *P*	N(%) Bonferroni *P*	N(%) Bonferroni *P*
Total	129	179(58.1)	90(29.2)	76(24.7)
Specialty		0.073	0.033	1.000
Internal Medicine	72	77(51.7)	32(21.5)	39(26.2)
Surgical Department	50	86(63.2)	49(36.0)	31(22.8)
Others	7	16(69.6)	9(39.1)	6(26.1)
Seniority (year)		0.192	0.777	0.579
< 5 or 7	63	74(54.0)	37(27.0)	30(21.9)
≥5 or 7	66	105(61.4)	53(31.0)	46(26.9)
Title		0.002	0.450	0.000
Junior	70	65(48.1)	38(28.1)	21(15.6)
Senior	45	99(68.8)	43(29.9)	49(34.0)
Advanced	14	15(51.7)	9(31.0)	6(20.7)
Work arrangement		0.734	1.000	1.000
Day-shift	28	36(56.3)	20(31.3)	15(23.4)
Rotating-shift	101	143(58.6)	70(28.7)	61(25.0)

We investigated whether the number of night shifts per month affected the prevalence of headache by grouping both doctors and nurses according to whether they worked more or less than the median of six (see Table 5). Doctors who worked more than six night-shifts were significantly more likely to suffer both types of headache than those who worked fewer than six night-shifts (migraine: 27.7% v.s. 21.2%; TTH: 27.7% v.s. 16.5%). Nurses who worked more than six night-shifts were significantly more likely to experience migraine headaches than those who worked fewer than six night-shifts (37.8% v.s. 18.8%); however, the prevalence of TTH did not significantly differ with the frequency of night shift work.

**Table 5:** The impact of number of night shifts per month on the prevalence of different types of headache among doctors and nurses

	Doctors						Nurses					
	Total headache	OR (95% CI)	Migraine	OR (95% CI)	TTH	OR (95% CI)	Total headache	OR (95% CI)	Migraine	OR (95% CI)	TTH	OR (95% CI)
	Prevalence (%)		Prevalence (%)		Prevalence (%)		Prevalence (%)		Prevalence (%)		Prevalence (%)	
Night shift number		1.94 (1.07–3.52)		1.80 (0.87–3.72)		2.31 (1.07–4.98)		1.67 (1.00–2.80)		2.72 (1.43–5.15)		0.99 (0.52–1.87)
< 6	34/85 (40.0)		18/85 (21.2)		14/85 (16.5)		61/117 (52.1)		22/117(18.8)		34/117(29.1)	
≥6	53/94 (56.4)		26/94 (27.7)		26/94 (27.7)		82/127 (64.6)		48/127(37.8)		27/127(21.3)	

The above factors were analysed by multivariate logistic regression (Tables 6 and 7), which showed that being female and working in other specialties remained independent risk factors for migraine in doctors. Female doctors were more than twice as likely as male doctors to experience migraine headaches, and doctors in other specialties were more likely to suffer migraines than doctors in the internal medicine and surgical departments. In nurses, being married and holding a senior job title remained independent risk factors for headaches overall, as did being married for migraine and having a senior job title for migraine and TTH. Nurses in the surgical department were more likely to suffer migraines than those in internal medicine. Nurses in other specialties were more likely to suffer migraines than internal medicine nurses, but there was no significant difference.

**Table 6:** Multivariable adjusted odds ratios (95% confidence interval) for total headache, migraine, and tension-type headache (TTH) among doctors

	Total headache	Migraine	TTH
Sex			
Male	Reference	Reference	Reference
Female	1.477(0.782–2.789)	2.314(1.005–5.326)*	1.088(0.493–2.402)
Specialty			
Internal Medicine	Reference	Reference	Reference
Surgical Department	1.364(0.720–2.586)	1.564(0.630–3.877)	1.275(0.586–2.771)
Others	1.932(0.814–4.586)	3.223(1.122–9.259)*	1.051(0.308–3.586)

*P < 0.05

**Table 7:** Multivariable adjusted odds ratios (95% confidence interval) for total headache, migraine, and tension-type headache (TTH) among nurses

	Total headache	Migraine	TTH
Marital status			
Unmarried/Divorced	Reference	Reference	Reference
Married	2.737(1.421–5.271)**	3.728(1.640–8.472)**	1.855(0.839–4.099)
Specialty			
Internal Medicine	Reference	Reference	Reference
Surgical Department	1.463(0.886–2.418)	1.910(1.036–3.521)*	1.018(0.536–1.936)
Others	2.180(0.797–5.962)	2.607(0.841–8.083)	1.352(0.375–4.876)
Title			
Junior	Reference	Reference	Reference
Senior	3.044(1.530–6.057)**	2.294(1.012–5.199)*	4.695(1.866–11.808)**
Advanced	1.720(0.527–5.616)	1.966(0.487–7.933)	2.006(0.422–9.524)

*P < 0.05, **P < 0.01

Among the doctors, sex and specialty were not risk factors for TTH in the multivariate logistic regression analysis, and age, nationality, marital status, education, BMI, seniority, title, and work arrangement were not identified as risk factors for either type of headache. Among the nurses, marital status and specialty were not identified as risk factors for TTH in the multivariate logistic regression analysis, and age, nationality, education, BMI, seniority, and work arrangement were not identified as risk factors for either type of headache.

## Discussion

Primary headache disorders are important causes of disability and reduced quality of life, and hospital workers are at high risk [21–23]. We found that the 1-year prevalence of primary headache disorders among medical staff in Sanya, South China, was 50% (migraine: 25.9%; TTH: 24.1%), which was significantly higher than the 23.8% (migraine: 9.3%; TTH: 10.8%) obtained in a population-based study in mainland China [3]. The prevalence rate of hospital workers in previous literature was high, as Sokolovic reported a 3-month prevalence of 61% [24], and Onwuekwe showed that the half-year prevalence was more than 70% [25]. There are two possible reasons for the difference. First, previous studies included non-clinical subjects (e.g., administrators and research staff). Second, a web-based survey adopted by previous studies may overrepresent persons with headache based on their willingness to respond.

Our nationwide population-based headache survey [3] suggested the prevalence of migraine was near the global average of 11%, while CDH (1% v.s. 3%) was less prevalent than it was worldwide. And MOH accounted for approximately 60% of CDH. Our group’s another study [26] showed MOH clinical profile -MOH in China was associated with lower educational level and annual income. A recent report [27] also suggested MOH was more common in the less well educated. Another study [15] recruited 1102 nurses among which only 10 respondents had two types of primary headache and only 2 were diagnosed with both CDH and MOH, suggesting nursing staff had lower prevalence of MOH and CDH than the general population.

Similar to their results, among 548 respondents in our survey only 3 respondents were diagnosed with two types of primary headache, and 18 had other headaches, including neuralgia, CDH, and unclassified headache. Only 1 nurse was diagnosed with MOH, which may be related to the group characteristics of medical staff. Generally medical staff have higher average education level and annual income than general population. Moreover, as a special occupational group with certain medical knowledge and convenient medical conditions, they took medicine more rationally and timely, which could be the main reason for the lower prevalence of MOH and CDH among medical staff.

As mentioned above, through headache questionnaires, face-to-face interviews and subsequent telephone follow-ups, we carefully confirmed only 3 respondents were diagnosed with two types of primary headache among 548 respondents, which was in accordance with the findings of the Chinese population-based door-to-door survey and northern nursing staff surveys [3, 15]. Of course, this may be associated with our small sample.

To the best of our knowledge, this study is the first to investigate the prevalence of primary headache disorders among doctors in mainland China (45% had primary headache disorder: 21.7% had migraine, and 23.3% had TTH). Unexpectedly, few data were available regarding headache among doctors on PubMed. Doctors’ headaches seemed to be ignored. Bartolini M reported that doctors were generally not more sensitised to problematic headache than the general population and tended to avoid evaluation by their general practitioner [28]. According to a survey of neurologists in Australia and New Zealand, 65.9% of neurologists had a personal history of migraine. This finding suggested significant under-recognition of migraine among non-neurologists [29]. In the future, headache disorder education should target interventions to raise awareness of cephalalgia disorders in populations of doctors.

When the prevalence of headache among nurses in Sanya, South China was analysed separately, we found a 53.9% prevalence of primary headache, including a 29.2% prevalence of migraine and a 24.7% prevalence of TTH. Compared with North China (where the prevalence rates were 45.3, 14.8, and 26.2% for primary headache, migraine and TTH, respectively) [15], the prevalence of TTH was similar, while migraine was more prevalent in South China. Geographical factors may be one explanation for the divergence. Sanya, located in the southernmost part of Hainan Island, is an international tourist city with a tropical maritime monsoon climate and plenty of sunlight. According to Tai MS, tropical weather can influence all types of trigger factors for migraine, and sunlight was a particularly common trigger factor in the equatorial and tropical regions of Asia [16]. Wang J reported that sunlight was one of the most common triggers for migraineurs in China [30].

Our study indicated that the prevalence of migraine in male doctors was lower than in female doctors. This result was consistent with the normal epidemiology of migraine, which was more common among women than among men [31]. However, female doctors seemed to have a higher prevalence of migraine than female nurses did. Chinese doctors must confront a large number of patients every day and perform scientific research to improve their knowledge. They were under great pressure and were very fatigued, which were triggers of migraine [32]. Strained doctor-patient relationships might also be responsible for this phenomenon.

Medical staff, a specific occupational group, were characterised by frequent shift work, especially night shifts. Night shift work had a series of well-known consequences, including sleep deprivation, sleep disturbances and irregular daily routines, which were considered triggers of migraine and TTH [33, 34]. The study in North China reported a higher prevalence of migraine among shift-working nurses [15]. Similar to previous findings, our results demonstrated that medical staff who worked a greater number of night shifts (≥6) had an increased prevalence of migraine. We found that working a relatively higher number of night shifts was associated with higher odds of TTH onset in doctors, but not in nurses. This phenomenon may be explained by the fact that doctors sit longer than nurses and that sitting causes an excessive forward head position or forward head posture (FHP). Fernández-de-Las-Peñas C found that FHP was associated with chronic tension-type headache (CTTH). His study suggested that shortened, contracted head and neck muscles associated with FHP may contribute to the development or perpetuation of TTH [35, 36]. However, further studies are required to investigate this possibility.

Multivariate analysis revealed that surgical nurses were more likely to have migraine than internal medicine nurses. Long and continuous working hours, missed meals, odours in the operating room and other common triggers of migraine may be responsible for the difference [37]. However, surgeons who worked in the same environment as the surgical nurses and experienced high stress levels during operations, were not more likely to have migraines than physicians. This may be attributable to a strong ability to manage stress and suggested that sex hormone may be a leading cause of migraine instead of other factors because almost all surgeons in China were male. In our study, a senior job title was significantly associated with a greater prevalence of headache (both TTH and migraine) in nurses, indicating that occupational factors affected the prevalence of headache. Nurses with senior titles tended to be married and had the dual responsibilities of mothers and wives. They must shoulder the burden of family and carry a high workload. In addition to clinical work, they were also responsible for management and teaching activities. However, the small number of participants with advanced titles may have affected the reliability of the results.

### Limitations

Our study had several limitations. First, the sample size was small, which may affect the accuracy of the results. It was not possible to further investigate the prevalence of and risk factors associated with subtypes of migraine and TTH. Second, the role of other factors, such as anxiety, depression, noise exposure, alcohol use and changes in the weather, which may potentially influence the prevalence of headache, should be explored in future studies. In addition, the recall bias arising from participants’ answers to occurrences and characteristics of headache in the past year may have affected the reliability of survey data, and prospective diary studies should be encouraged.

## Conclusion

Our study shows that the prevalence of primary headache disorders (including migraine and TTH) in both nurses and doctors is higher than that of the general population in South China. The special geographical location of Sanya could be responsible for the difference in the prevalence of migraine between nurses in North and South China. Multiple factors associated with migraine and TTH among medical staff were verified, including sex and specialty for migraine in doctors; marital status, title and specialty for migraine in nurses; and job title for TTH in nurses. To improve the health of medical staff, headache education and strategies for managing these factors should be effectively implemented.

## Data Availability

The datasets used and analyzed during the current study are available from the corresponding author on reasonable request.

## Abbreviations

CDH: Chronic daily headache
ICHD-3: International Classification of Headache Disorders, 3rd edition
MOH: Medication overuse headache
TTH: Tension type headache
